# Antibiofilm and antibacterial activity of maslinic acid against Vancomycin-Resistant Enterococci (VRE)

**DOI:** 10.1371/journal.pone.0342234

**Published:** 2026-02-10

**Authors:** Somaia M. Abdelmegeed, Mohamed F. Mohamed, Mohamed N. Seleem

**Affiliations:** 1 Department of Biomedical Sciences and Pathobiology, Virginia-Maryland College of Veterinary Medicine, Virginia Polytechnic Institute and State University, Blacksburg, Virginia, United States of America; 2 Center for One Health Research, Virginia Polytechnic Institute and State University, Blacksburg, Virginia, United States of America; 3 Department of Bacteriology, Mycology and Immunology, Faculty of Veterinary Medicine, Beni-Suef University, Beni Suef, Egypt; Yakin Dogu Universitesi, TÜRKIYE

## Abstract

The emergence of vancomycin-resistant *Enterococcus* (VRE) has posed a significant global health threat, especially in healthcare settings where treatment options are increasingly limited due to rising antibiotic resistance. Maslinic acid, a naturally occurring pentacyclic triterpene found in olives, is known for its diverse biological activities, including anti-inflammatory, anticancer, antioxidant, and antimicrobial effects. In this study, we investigated the antibacterial and antibiofilm potential of maslinic acid against VRE. Initial screening identified maslinic acid as a potent hit, with minimum inhibitory concentrations (MICs) ranging from 4 to 8 µg/mL across 13 clinical enterococcal isolates, including multidrug-resistant strains. Time-kill assays demonstrated bacteriostatic activity comparable to linezolid, while cytotoxicity and hemolysis assays confirmed its safety in mammalian cells. Furthermore, maslinic acid disrupted established *Enterococcus faecalis* biofilms by approximately 50%, whereas linezolid was not effective against biofilms. Notably, maslinic acid significantly reduced bacterial burden in a *Caenorhabditis elegans* infection model by 80%, outperforming linezolid. These findings highlight maslinic acid as a promising candidate for the development of new therapies targeting VRE, with the added advantage of antibiofilm activity and a favorable safety profile.

## Introduction

Vancomycin-resistant *Enterococcus* (VRE) has emerged as a major global health concern, particularly in healthcare settings, where it is responsible for persistent and life-threatening infections [1]. *Enterococcus* species, including *Enterococcus faecium* and *Enterococcus faecalis*, are typically harmless commensals in the human gastrointestinal tract. However, due to their ability to acquire and disseminate resistance genes, these bacteria have become formidable pathogens, posing significant therapeutic challenges [2]. The emergence of resistance to vancomycin, a last-resort antibiotic for treating Gram-positive bacterial infections, has severely limited treatment options [3–5]. VRE infections are associated with high morbidity and mortality rates, particularly in immunocompromised patients, making the development of novel antimicrobial agents a critical priority [2].

Managing enterococcal infections presents considerable difficulty due to extensive antibiotic resistance. VRE, in particular, have emerged as a critical public health threat [2]. Data from the Centers for Disease Control and Prevention (CDC) indicate that VRE accounted for around 55,000 hospitalizations in the United States in 2017, with a mortality rate of 10% and an estimated healthcare cost burden of $540 million [6,7]. This issue is intensified by VRE’s capacity to swiftly develop and disseminate resistance mechanisms through genetic mutations and horizontal gene transfer. In recognition of these challenges, the World Health Organization (WHO) has designated VRE as a high-priority multidrug-resistant pathogen, underlining the urgent need for new treatment strategies [8].

The available treatment options for VRE infections remain scarce. Linezolid is the only antibiotic sanctioned by the FDA specifically for the treatment of VRE. Despite its approval, several clinical issues limit its efficacy, including a high mortality rate—particularly in bloodstream infections— poor efficacy in eliminating gastrointestinal colonization, and notable adverse effects, such as bone marrow suppression and neurotoxicity [9,10]. Although daptomycin is often used in practice, it does not carry FDA approval for VRE, compounded by issues of inconsistent dosing regimens [11]. Moreover, resistance to multiple first-line antibiotics—including quinupristin/dalfopristin, daptomycin, linezolid, and tigecycline—has been increasingly reported, underscoring the critical demand for novel and potent treatment options [3–5].

Due to the alarming spread of multidrug-resistant bacteria, researchers are actively investigating alternative therapeutic strategies, especially bioactive substances derived from nature. Natural products have long served as an essential reservoir for antimicrobial discovery, offering compounds with diverse structures and unique mechanisms of action. One such candidate is maslinic acid, a pentacyclic triterpene predominantly naturally present in olives, and noted for its diverse biological activities, including anti-inflammatory, anticancer, antioxidant, and antimicrobial effects [12,13]. While its efficacy exhibited inhibitory effects on a range of Gram-positive bacterial strains, there is limited information regarding its impact on VRE. Investigating the antimicrobial properties of maslinic acid against resistant pathogens may pave the way for the development of innovative treatment alternatives.

A major challenge in treating VRE infections is their ability to form biofilms, which shield the bacteria from both the host’s immune system and antimicrobial treatments, making these infections particularly hard to eliminate [14]. Biofilms significantly contribute to the chronic nature of VRE infections, especially in cases involving catheter-associated urinary tract infections, endocarditis, and bloodstream infections [15]. Therefore, effective treatment strategies must target not only bacterial growth but also the biofilm structures that facilitate persistent infections.

This study aims to investigate the antibacterial and antibiofilm properties of maslinic acid against VRE using a comprehensive experimental approach. The antimicrobial activity was assessed by determining the minimum inhibitory concentration (MIC) against 13 clinical VRE isolates. To evaluate the safety of maslinic acid, cytotoxicity was tested using Caco-2 cells. A time-kill assay was conducted to determine whether the compound has bacteriostatic or bactericidal effects. Additionally, a biofilm eradication assay was used to evaluate its ability to disrupt established biofilms. Finally, an *in vivo* infection model using *Caenorhabditis elegans* was employed to assess the therapeutic efficacy of maslinic acid in a living host.

Through this investigation, this study aims to provide novel insights into the antimicrobial properties of maslinic acid and its potential as a therapeutic agent against VRE infections. The findings from this research may contribute to the ongoing efforts to develop alternative treatments for multidrug-resistant bacterial infections, ultimately aiding in the global fight against antibiotic resistance.

## Materials and methods

### Bacterial strains, chemicals, and culture medium

All bacterial culture media and chemical reagents used in this study were obtained from commercial vendors. *Enterococcus* strains were acquired from the American Type Culture Collection (ATCC, Manassas, VA, USA) and the Biodefense and Emerging Infections Research Resources Repository (BEI Resources, Manassas, VA, USA). The maslinic acid-based pentacyclic triterpene focused library (Cat. No. HY-L0001) was purchased from MedChemExpress (Monmouth Junction, NJ, USA). Linezolid was obtained from Selleck Chemicals (Houston, TX, USA), while vancomycin hydrochloride was acquired from Gold Biotechnology (St Louis, MO, USA). Tryptic soy agar (TSA) and tryptic soy broth (TSB) were sourced from Becton, Dickinson (Cockeysville, MD). Caco-2 cells (a cell line derived from human colorectal adenocarcinoma), were acquired from the American Type Culture Collection (ATCC, Manassas, VA, USA).

### Screening a maslinic acid-based library for antimicrobial activity against enterococci

The maslinic acid-based pentacyclic triterpene-focused library (a panel of 13 natural maslinic acid-derived compounds) was screened against *E. faecium* NR-31909 to determine antimicrobial efficacy of the compounds. The assay followed Clinical and Laboratory Standards Institute (CLSI) guidelines [16,17] with minor modifications. Briefly, *E. faecium* NR-31909 cultures were initiated on TSA and incubated at 37°C for 24 hours. Colonies were suspended in TSB and adjusted to 0.5 McFarland turbidity standard, yielding a final inoculum of approximately 5 × 10⁵ CFU/mL. The bacterial suspension was dispensed into 96-well microtiter plates containing test compounds at a final concentration of 64 µM. Plates were incubated at 37°C for 24 hours. Dimethyl sulfoxide (DMSO) served as the negative control, and vancomycin and linezolid were included as reference standards. Bacterial growth inhibition was assessed using an a microplate reader (Synergy H1, BioTek, USA) at OD_600_, and minimum inhibitory concentrations (MICs) were determined via broth microdilution. MIC was defined as the lowest concentration with complete inhibition of visible growth [18].

### Antibacterial activity of the maslinic acid against various clinical enterococci strains

To further investigate its antibacterial potential, maslinic acid—selected for its potent activity against vancomycin-resistant *Enterococcus*—was subjected to additional testing. A commercially available source of maslinic acid (Ambeed, IL, USA) was evaluated against a panel of 13 clinical *Enterococcus* isolates. Antibacterial efficacy was compared to standard-of-care antibiotics, vancomycin and linezolid, using the broth microdilution method as previously described [16,17]. MIC values were determined to assess relative potency across the strain panel [18]..

### Minimum Bactericidal Concentration (MBC)

The MBC of the tested compounds against enterococci strains was determined using methods described previously [19]. Aliquots (4 μL) of enterococci strains were transferred to TSA plates, Plates were incubated at 37°C for 24 hrs before the MBC, the concentration where >99% reduction in bacterial cell count was observed, was determined.

### Time-kill assay

The time-dependent antibacterial activity of maslinic acid was assessed using a time-kill assay, adapted from our previous study [18] and established protocols [20]. *E. faecium* NR-31909 was cultured overnight in TSB, subcultured into fresh TSB, and incubated at 37°C under aerobic conditions until reaching mid-log phase (OD₆₀₀ ≈ 0.2). The cultures were then diluted to a final concentration of 5 × 10⁵ CFU/mL. Maslinic acid and linezolid were tested at 5 × MICs (20 µg/mLfor maslinic acid and 2.5 µg/mL for linezolid) and 10 × MICs (40 µg/mL for maslinic acid and 5 µg/mL for linezolid). A DMSO-treated culture was used as negative control. Cultures were incubated at 37°C with shaking, and aliquots were collected at defined time points (2, 4, 6, 8 and 24 hrs). Samples were serially diluted in phosphate-buffered saline (PBS) and plated in triplicate on TSA. After 24 hours of incubation at 37°C, colony-forming units (CFUs) were enumerated.

### Cytotoxicity assessment in Caco-2 cells

The cytotoxic potential of maslinic acid was evaluated in Caco-2 cells following previously described procedures [18,21]. Cells were seeded into 96-well plates containing Eagle’s Minimum Essential Medium (EMEM) supplemented with 10% fetal bovine serum (FBS) and incubated at 37°C in a humidified atmosphere with 5% CO₂ for 24 hours to allow for adherence and growth.

After incubation, the medium was replaced with fresh EMEM containing serial dilutions of maslinic acid. Wells treated with solvent alone (DMSO) served as the negative control. Treated cells were incubated for an additional 24 hours under the same conditions. Cell viability was then assessed using the 3-(4,5-dimethylthiazol-2-yl)-5-(3-carboxymethoxyphenyl)-2-(4-sulfophenyl)-2H-tetrazolium(MTS)/ Phenazine Methyl Sulphate (PMS) (MTS/PMS) assay. Reagents were added to each well and incubated for four hours, after which absorbance at 490 nm was measured using a microplate reader (Synergy H1, BioTek Instruments, USA). All assays were performed in triplicate using biologically independent replicates.

### Hemolysis assay

The hemolytic potential of maslinic acid was assessed using human red blood cells (RBCs), following previously published protocols with minor modifications [18,22,23]. Freshly collected human RBCs were centrifuged at 2,000 rpm for 5 minutes to obtain a cell pellet, which was then washed three times with phosphate-buffered saline (PBS).

A working suspension of 8% (v/v) RBCs was prepared in PBS, and 50 µL of this suspension was dispensed into each well of a 96-well plate. Equal volumes (50 µL) of maslinic acid solutions at various concentrations, prepared in PBS, were added to the wells to achieve a final RBC concentration of 4% (v/v). PBS alone was used as the negative control, while 0.1% Triton X-100 served as the positive (100% hemolysis) control.

The plate was incubated at 37°C for 1 hour, followed by centrifugation at 1,000 RPM for 5 minutes at 4°C to pellet intact RBCs. A 75 µL aliquot of the supernatant from each well was carefully transferred to a new 96-well plate. Hemoglobin release, as an indicator of hemolysis, was quantified by measuring absorbance at 405 nm using a microplate reader (Synergy H1, BioTek Instruments, USA). The percentage of hemolysis was calculated relative to the positive control. All measurements were performed in three independent biological replicates.

### Evaluation of maslinic acid on bacterial biofilms

The efficacy of maslinic acid in disrupting bacterial biofilms was assessed using a microtiter plate biofilm formation assay, based on established protocols [21,22]. Culture of *E. faecalis* NR-31887 was grown overnight in TSB. Overnight cultures were diluted 1:100 in biofilm growth medium, (TSB + 1% glucose) to support biofilm development. Biofilms were allowed to develop for 24 hours incubation. After the initial incubation period, wells were gently washed twice with PBS to remove any non-adherent planktonic cells. Fresh medium containing the test compounds—maslinic acid,linezolid or DMSO as negative control—was then added, and the plates were incubated for an additional 24 hours. Following this second incubation, the wells were washed three times with distilled water to eliminate residual medium and unattached cells. The biofilms were air-dried and stained using a 0.1% crystal violet solution for 30 minutes to visualize the adherent biomass. Excess crystal violet was removed by washing with distilled water, and the remaining dye bound to biofilms was solubilized using 30% glacial acetic acid. The extent of biofilm formation and disruption was quantified by measuring absorbance at 550 nm using a microplate reader (Synergy H1, BioTek, USA). To ensure accuracy and reproducibility, each experiment was performed in three biological replicates and repeated independently twice.

### *In vivo* efficacy of maslinic acid in a *Caenorhabditis elegans* (*C. elegans*) infection model

The *Caenorhabditis elegans* temperature-sensitive sterile mutant strain AU37 [sek-1(km4); glp-4(bn2) I] was utilized to assess the *in vivo* antimicrobial efficacy of maslinic acid, following established protocols [22,24–27]. Adult worms were initially cultured on nematode growth medium (NGM) agar plates seeded with *Escherichia coli* OP50 at 15°C for 5 days to promote egg laying. Eggs were harvested via bleaching and incubated at room temperature with gentle agitation for 24 hours to allow hatching. Newly hatched larvae were transferred to fresh NGM plates containing *E. coli* OP50 and maintained at room temperature until reaching adulthood. Mature worms were collected and washed three times with PBS to remove residual bacteria before infection with *E. faecium* NR-31909. Following infection, worms were again washed thrice with PBS. Approximately 30 worms per treatment group were then exposed to maslinic acid or linezolid at 10 × MIC for 24 hours. To quantify bacterial load, worms were rinsed three times with PBS, transferred to tubes containing 200 mg of 1.0-mm silicon carbide beads (Biospec Products, Bartlesville, OK, USA), and lysed by vortexing vigorously for 5 minutes. The resulting homogenate was plated onto TSA supplemented with ampicillin and vancomycin to select for vancomycin-resistant *Enterococcus* (VRE). Plates were incubated at 37°C for 24 hours, and bacterial colonies were enumerated.

## Results

### Screening of maslinic acid library against *Enterococcus*

To explore novel therapeutic options against enterococcal infections, a panel of 13 natural maslinic acid-derived compounds was evaluated for activity against vancomycin-resistant *E. faecium* NR-31909. Each compound was initially tested at a concentration of 64 μM. All candidates showed inhibitory effects on bacterial growth at the screening concentration, suggesting promising antimicrobial potential. MICs for the most active compounds ranged between 8 and 32 μM (Table 1). Based on its potent activity, maslinic acid was selected for more detailed investigation.

**Table 1:** Description and MICs of active compounds of Maslinic acid library against *E. faecium* NR-31909.

Compound	MIC (µM)	MW (g/mol)	MIC (µg/mL)
Maslinic acid	8	472.7	3.8
Corosolic acid	16	472.7	7.6
Pomolic acid	16	472.7	7.6
Ursonic acid	32	454.6	14.5
3-Epiursolic acid	32	456.7	14.6
Echinocystic acid	32	472.7	15.1
3-Epioleanolic acid	32	456.7	14.6
Ursolic acid	16	456.7	7.3
Asiatic acid	32	488.7	15.6
Epibetulinic acid	32	456.7	14.6
β-Boswellic acid	32	456.7	14.6
Oleanolic acid	32	456.7	14.6
Polygalacic acid	32	488.7	15.6
Linezolid	0.5	337.35	0.17
Vancomycin	>64	1449	>92.7

### Antibacterial activity of maslinic acid against clinical *Enterococcus* isolates

The antimicrobial efficacy of maslinic acid was further evaluated using a panel of 13 clinical *Enterococcus* isolates, comprising both vancomycin-resistant and susceptible strains of *E. faecium*, *E. faecalis*, and *E. durans* (Table 2). Maslinic acid exhibited MICs ranging from 4 to 8 μg/mL against all tested strains, with the exception of *E. durans* ATCC 11576, which showed high susceptibility (MIC < 1 μg/mL). In comparison, MIC values for linezolid ranged from 0.25 to 1 μg/mL, whereas most isolates demonstrated resistance to vancomycin (>64 µg/mL).

**Table 2:** MIC values (μg/mL) of the Maslinic acid and control antibiotics against clinical isolates of enterococci.

Strain	Maslinic acid	Linezolid	Vancomycin
*Enterococcus faecium* NR-31909	4	0.5	>64
*Enterococcus faecium* NR 31912	4	0.5	>64
*Enterococcus faecium* NR-31903	4	0.5	>64
*Enterococcus faecium* - E417	8	0.5	>64
*Enterococcus faecium* - ERV165	8	1	>64
*Enterococcus faecium* - UAA945	8	1	>64
*Enterococcus faecium* - HF50104	4	0.5	>64
*Enterococcus faecium* – 503	8	0.5	>64
*Enterococcus faecalis* NR31972	8	1	>64
*Enterococcus faecalis* - B3336	8	0.5	>64
*Enterococcus faecalis* - S613	8	1	>64
*Enterococcus faecalis* - R712	8	1	>64
*Enterococcus durans* ATCC 11576	<1	0.25	<1

### Minimum Bactericidal Concentration (MBC)

Maslinic acid demonstrated primarily bacteriostatic activity against *Enterococcus* species, as MBC values were substantially higher than the corresponding MICs (4–8 µg/mL) (Table 3). For most *E. faecium* and *E. faecalis* isolates, MBCs were ≥64 µg/mL or exceeded the highest concentration tested (>128 µg/mL). Limited bactericidal activity was observed in a small number of strains, including *E. faecium* NR-31909 and E417 (MBC = 32 µg/mL) and *E. faecalis* B3336 (MBC = 16 µg/mL. Although *Enterococcus durans* ATCC® 11576 was highly susceptible (MIC < 1 µg/mL), its MBC (4 µg/mL) exceeded the MIC, indicating a bacteriostatic effect. Overall, the large MBC/MIC ratios indicate that maslinic acid acts predominantly as a bacteriostatic agent against *Enterococcus* spp.

**Table 3:** MBC (μg/mL) of Maslinic acid against enterococci strains.

Strain	MBC (μg/mL)
*Enterococcus faecium NR-31909*	32
*Enterococcus faecium NR-31912*	>128
*Enterococcus faecium NR-31903*	>128
*Enterococcus faecium - E417*	32
*Enterococcus faecium - ERV165*	>128
*Enterococcus faecium - UAA945*	128
*Enterococcus faecium - HF50104*	>128
*Enterococcus faecium – 503*	>128
*Enterococcus faecalis NR31972*	>128
*Enterococcus faecalis - B3336*	16
*Enterococcus faecalis - S613*	64
*Enterococcus faecalis - R712*	64
*Enterococcus durans ATCC 11576*	4

### Time kill kinetics

**Fig 1:**
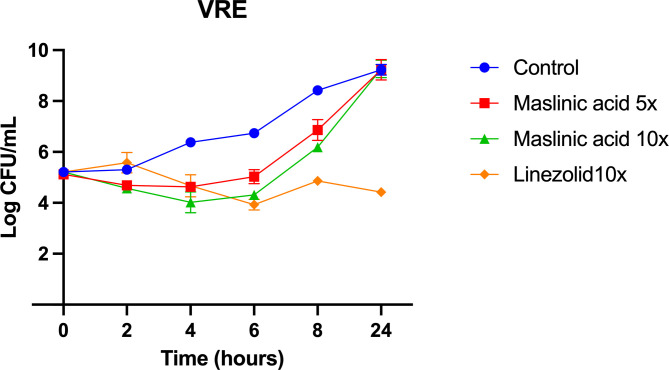
Time kill assay of maslinic acid at 5 × and 10 × MIC and linezolid at 10 × MIC, against *E. faecium* NR-31909. Samples treated with DMSO were used as negative control. The results are given as means ± SD (n = 3; data without error bars indicate that the SD is too small to be seen).

To assess whether maslinic acid exerts bacteriostatic or bactericidal effects against *Enterococcus*, a time-kill kinetics assay was performed using *E. faecium* NR-31909. As illustrated in Fig 1, maslinic acid at concentrations of 5× and 10 × its MIC demonstrated bacteriostatic activity, showing growth inhibition comparable to that observed with linezolid.

### Cytotoxicity assessment in vero cells

**Fig 2:**
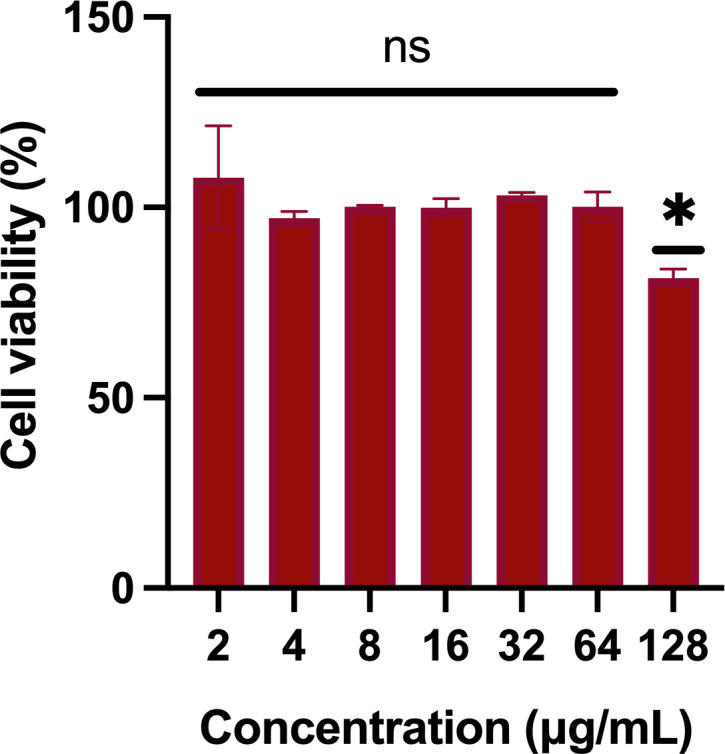
Cytotoxicity assay showing the percent mean absorbance at 490 nm after incubating Caco-2 cells with maslinic acid at different concentrations for 24 hr. Cell viability was measured by MTS assay. Results are expressed as means from three measurements ± standard deviations. All experiments were done in triplicate. Statistical analyses were determined by one-way ANOVA with post hoc testing (*p < 0.05). ns, non-significant.

To evaluate the potential cytotoxic effects of maslinic acid, assays were conducted using Caco-2 cells. The compound exhibited no observable toxicity at concentrations up to 64 µg/mL, with a calculated IC₅₀ value of 128 µg/mL (Fig 2).

### Hemolytic activity evaluation

**Fig 3:**
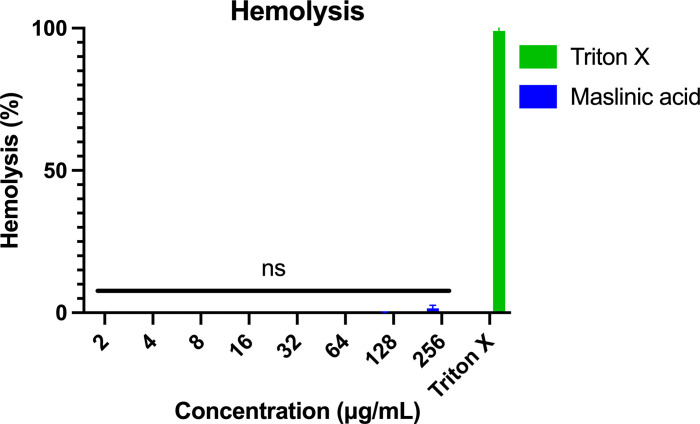
Hemolysis assay of maslinic acid on human RBCs. The release of hemoglobin in the supernatant of human erythrocytes after treatment with increasing amounts of the two compounds was measured at 405 nm. Data collected after 1 h of incubation are presented. 0.1% of Triton X-100 served as positive control. All experiments were done in triplicate. Statistical analyses were determined by one-way ANOVA with post hoc testing. ns, non-significant.

A hemolysis assay was performed to evaluate the potential of maslinic acid to lyse human RBCs. No hemolytic activity was observed at concentrations up to 256 µg/mL, the highest dose tested (Fig 3). These findings suggest that maslinic acid possesses a favorable safety profile and exhibits strong selectivity for bacterial cells over mammalian cells.

### Evaluation of maslinic acid on bacterial biofilms

**Fig 4:**
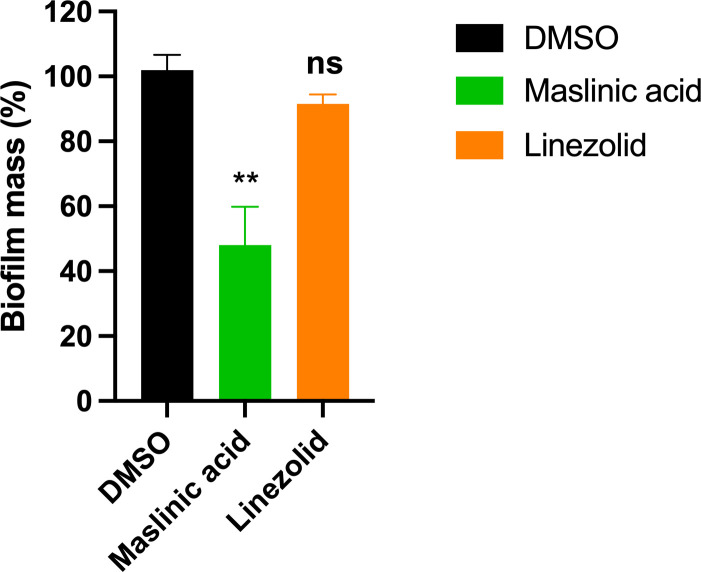
The effect of maslinic acid and linezolid on the biofilms of *E. faecalis* NR-31887. The adherent biofilm was stained by crystal violet, then the dye was extracted with ethanol, measured at 595 nm absorbance and presented as percentage of biofilm reduction compared to untreated wells. All experiments were done in triplicate. Statistical analyses were determined by one-way ANOVA with post hoc testing (**p < 0.01). ns, non-significant.

Maslinic acid significantly reduced the biomass of preformed *E. faecalis* NR-31887 biofilms. After 24 hours of treatment, crystal violet staining revealed a marked decrease in biofilm density compared to the untreated control. Quantification by absorbance at 550 nm showed that maslinic acid at 8 × MIC resulted in a 50% reduction in biofilm biomass (*p* < 0.05) (Fig 4). In contrast, linezolid treatment resulted in only a slight, statistically non-significant decrease in biofilm biomass (*p* > 0.05), indicating a limited effect on established biofilms (Fig 4). These results highlight the superior antibiofilm activity of maslinic acid over linezolid against *E. faecalis* NR-31887.

### *In Vivo* Efficacy of maslinic acid in a *C. elegans* infection model

**Fig 5:**
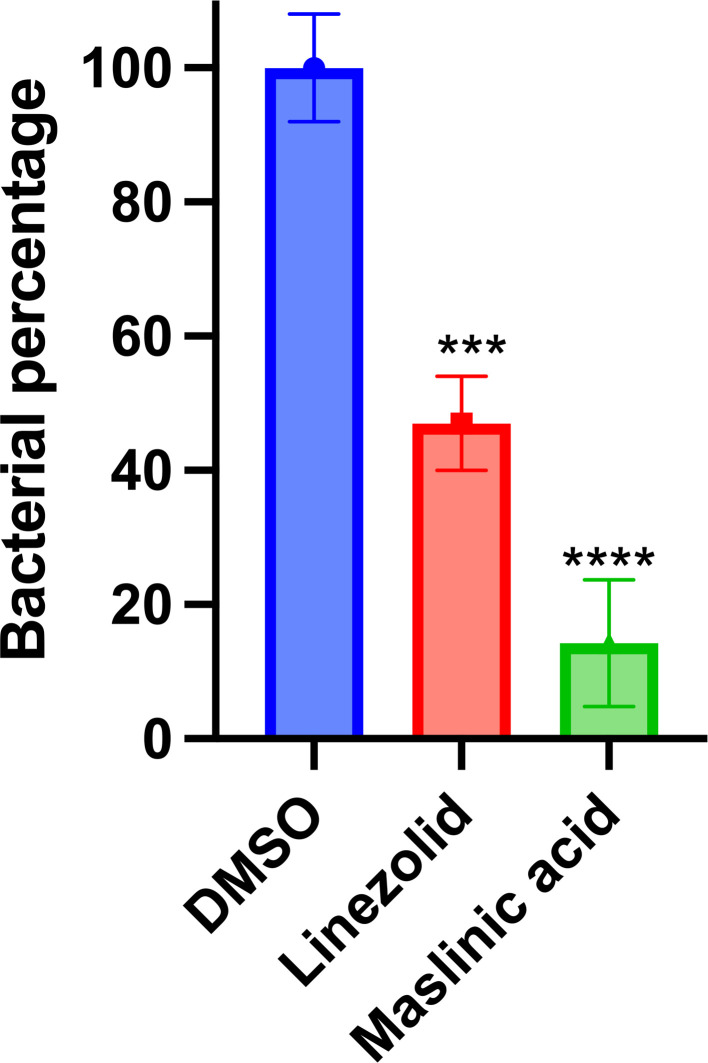
*In vivo* efficacy of maslinic acid in a *Caenorhabditis elegans* model of bacterial infection. *C. elegans* were infected with VRE*, E. faecium* NR-31909. After infection, worms were treated with maslinic acid or linezolid at 10 × MIC. After 24 hours, worms were lysed, and bacteria were plated and CFU were counted after 24 hr. Results are expressed as means from three biological replicates ± standard deviation. Statistical analyses were determined by one-way ANOVA with post hoc testing (***p < 0.005), (****p < 0.0001).

To validate the *in vivo* antimicrobial activity of maslinic acid, a *C. elegans* infection model was employed. Worms were infected with *E. faecium* NR-31909 and subsequently treated with maslinic acid or linezolid at 10 × their respective MICs. After 24 hours of treatment, bacterial burden was quantified following worm lysis. As shown in Fig 5, maslinic acid treatment resulted in an 80% reduction in VRE load (*p* < 0.0001), compared to a 55% reduction observed with linezolid (*p* < 0.0001). These findings highlight the strong *in vivo* efficacy of maslinic acid against VRE.

## Discussion

VRE infections continue to pose a serious clinical threat, driven by the growing resistance to critical last-resort antibiotics such as linezolid and daptomycin [2]. This rising resistance underscores the urgent need for new antimicrobial agents with novel mechanisms of action to effectively address these difficult-to-treat infections [4]. This study provides compelling evidence supporting the antimicrobial potential of maslinic acid, a naturally occurring triterpenoid compound abundantly found in the skins of olives. Through a comprehensive evaluation of *in vitro* and *in vivo* models, maslinic acid demonstrated promising activity against *Enterococcus* spp., including multidrug-resistant strains, and effectively disrupted established biofilms, a critical virulence trait contributing to chronic and recurrent enterococcal infections.

Our initial screening of maslinic acid derivatives revealed inhibition of *E. faecium* NR-31909, a vancomycin-resistant isolate, at micromolar concentrations. The purified maslinic acid compound exhibited consistent activity across a panel of 13 clinical isolates, with MIC values ranging from 4 to 8 µg/mL for most strains. These findings underscore maslinic acid’s capacity to inhibit both vancomycin-resistant and susceptible *E. faecium* and *E. faecalis* strains. Compared to standard antibiotics, vancomycin showed limited efficacy against most isolates tested, while linezolid exhibited expected potency but lacked significant antibiofilm activity.

Time-kill assays confirmed the bacteriostatic nature of maslinic acid, as bacterial growth was suppressed without significant killing over the observed time period. Importantly, maslinic acid was non-toxic to mammalian Caco-2 cells up to 64 µg/mL and showed no hemolytic activity at concentrations as high as 256 µg/mL, indicating a strong safety profile and selective activity toward bacterial cells.

One of the most significant outcomes of this study was the observation that maslinic acid substantially disrupted mature *E. faecalis* biofilms. Biofilms are central to the persistence and antibiotic resistance of enterococcal infections [28,29]. While linezolid exhibited negligible effects on preformed biofilms, maslinic acid at 8 × MIC reduced biofilm biomass by approximately 50%, suggesting a unique capability to penetrate and destabilize biofilm matrices. This antibiofilm activity may provide an important therapeutic advantage, especially in device-associated infections or environments where enterococci persist in biofilm communities [28,29].

The *C. elegans* infection model provided further insight into the *in vivo* efficacy of maslinic acid. Treatment with maslinic acid led to an 80% reduction in bacterial load, outperforming linezolid, which achieved a 55% reduction. These findings support the translational relevance of maslinic acid as a therapeutic candidate with demonstrable activity in a living host model.

Several pentacyclic triterpenes, including ursolic acid and betulinic acid, have been reported to exhibit antibacterial activity against Gram-positive bacteria. Betulinic acid has been reported to exhibit antibacterial activity against selected Gram-positive bacteria, including *Staphylococcus aureus*, with mechanistic studies suggesting enzyme-targeted modes of action [30]. Ursolic acid has demonstrated inhibitory and antibiofilm effects primarily against *Staphylococcus aureus* and related pathogens, while its activity against *Enterococcus* species and vancomycin-resistant strains has been less extensively explored [31]. Notably, when these triterpenes were evaluated side by side against *E. faecium* in our initial screening, maslinic acid exhibited the strongest antibacterial activity, displaying the lowest MIC among all compounds tested.

Although the precise mechanism of action of maslinic acid was not directly investigated in this study, several plausible mechanisms can be proposed. As a pentacyclic triterpene with amphipathic properties, maslinic acid may interact with bacterial membranes, leading to altered membrane organization or permeability without causing rapid cell lysis, consistent with its bacteriostatic activity [30]. In addition, triterpenes have been reported to interfere with cell wall–associated processes, including membrane-associated enzymes essential for bacterial growth [12,30].

The pronounced ability of maslinic acid to disrupt established *Enterococcus* biofilms further suggests a role in perturbing biofilm matrix integrity or interfering with surface-associated regulatory pathways that maintain biofilm structure [14,15]. Together, these observations indicate that maslinic acid may exert its antibacterial effects through multi-target interactions rather than a single lethal mechanism, which could contribute to its activity against multidrug-resistant enterococci.

While this study demonstrates the antibacterial and antibiofilm activity of maslinic acid, certain limitations should be acknowledged. Although the *C. elegans* infection model provides a useful in vivo platform for evaluating antimicrobial efficacy, it does not fully capture the complexity of mammalian host–pathogen interactions. Consequently, the absence of a mammalian infection model limits conclusions regarding pharmacokinetics, tissue distribution, immune modulation, and therapeutic dosing. Future studies employing mammalian models will be essential to further validate the efficacy and safety of maslinic acid and to support its translational development.

The natural origin of maslinic acid, particularly its abundance in olive skin, invites further exploration into the dietary and preventive health implications of olive consumption. It is plausible that regular intake of olives or olive-derived products could support microbiota modulation or reduce intestinal colonization by resistant enterococci [32–34]. While dietary concentrations of maslinic acid are unlikely to match therapeutic levels used in this study, they may still contribute to colonization resistance, especially when combined with other antimicrobial dietary components.

In conclusion, maslinic acid emerges as a promising natural compound with potent antibacterial and antibiofilm properties against multidrug-resistant enterococci. Its favorable safety profile, broad-spectrum activity, and *in vivo* efficacy make it a strong candidate for further preclinical development. Moreover, the potential health benefits associated with dietary sources of maslinic acid merit additional investigation into its role in infection prevention and microbiome modulation.

## Supporting information

S1 File Raw data figures, contain all raw data sets for figures in the manuscript. (XLSX)
